# Early pregnancy sex steroids during primiparous pregnancies and maternal breast cancer: a nested case–control study in the Northern Sweden Maternity Cohort

**DOI:** 10.1186/s13058-017-0876-8

**Published:** 2017-07-18

**Authors:** Renée T. Fortner, Eglé Tolockiene, Helena Schock, Husam Oda, Hans-Åke Lakso, Göran Hallmans, Rudolf Kaaks, Paolo Toniolo, Anne Zeleniuch-Jacquotte, Kjell Grankvist, Eva Lundin

**Affiliations:** 1 Division of Cancer Epidemiology, German Cancer Research Center (DKFZ), Im Neuenheimer Feld 280, D-69120 Heidelberg, Germany; 2 Department of Medical Biosciences, Pathology, Umeå University, Umeå, Sweden; 3 Department of Medical Biosciences, Clinical Chemistry, Umeå University, Umeå, Sweden; 4 Public Health and Clinical Medicine: Nutritional Research, Umeå University, Umeå, Sweden; 5 Department of Obstetrics and Gynecology, New York University School of Medicine, New York, NY USA; 6 Department of Population Health and Perlmutter Cancer Center, New York University School of Medicine, New York, NY USA

**Keywords:** Endogenous hormones, Early pregnancy, Breast cancer, Sex steroids

## Abstract

**Background:**

Pregnancy and parity are associated with subsequent breast cancer risk. Experimental and epidemiologic data suggest a role for pregnancy sex steroid hormones.

**Methods:**

We conducted a nested case–control study in the Northern Sweden Maternity Cohort (1975–2007). Eligible women had provided a blood sample in the first 20 weeks of gestation during a primiparous pregnancy leading to a term delivery. The current study includes 223 cases and 417 matched controls (matching factors: age at and date of blood collection). Estrogen receptor (ER) and progesterone receptor (PR) status was available for all cases; androgen receptor (AR) data were available for 41% of cases (*n* = 92). Sex steroids were quantified by high-performance liquid chromatography tandem mass spectrometry. Odds ratios (ORs) and 95% confidence intervals were estimated using conditional logistic regression.

**Results:**

Higher concentrations of circulating progesterone in early pregnancy were inversely associated with ER+/PR+ breast cancer risk (OR_log2_: 0.64 (0.41–1.00)). Higher testosterone was positively associated with ER+/PR+ disease risk (OR_log2_: 1.57 (1.13–2.18)). Early pregnancy estrogens were not associated with risk, except for relatively high estradiol in the context of low progesterone (split at median, relative to low concentrations of both; OR: 1.87 (1.11–3.16)). None of the investigated hormones were associated with ER–/PR– disease, or with AR+ or AR+/ER+/PR+ disease.

**Conclusions:**

Consistent with experimental models, high progesterone in early pregnancy was associated with lower risk of ER+/PR+ breast cancer in the mother. High circulating testosterone in early pregnancy, which likely reflects nonpregnant premenopausal exposure, was associated with higher risk of ER+/PR+ disease.

**Electronic supplementary material:**

The online version of this article (doi:10.1186/s13058-017-0876-8) contains supplementary material, which is available to authorized users.

## Background

Pregnancy is associated with a transient increase in risk of breast cancer for up to a decade post delivery, and subsequently confers a protective effect for women younger than 30 at first birth and for estrogen receptor (ER) and progesterone receptor (PR) positive tumors [1]. Animal models suggest that pregnancy-associated increases in estradiol and progesterone confer a protective effect against breast cancer [2] and it is hypothesized that higher concentrations of circulating estradiol and progesterone during pregnancy may be associated with the long-term decreased risk of breast cancer in women.

Endogenous sex steroid hormones and breast cancer risk outside of pregnancy have been extensively investigated in both premenopausal [3, 4] and postmenopausal [5–7] women, with the majority of data for women postmenopausal at blood collection. However, to date, the association between endogenous sex steroids in pregnancy and breast cancer risk in the mother has been investigated in only two populations [8–10]. Results from the largest study to date, a case–control study nested within the Finnish Maternity Cohort (FMC), suggest that the association between early pregnancy endogenous hormones and breast cancer may differ depending on age at diagnosis. In the FMC, higher concentrations of sex steroids were associated with higher risk among women diagnosed at relatively young age (<40 years), and predominantly for ER–/PR– disease, while inverse associations were observed with higher estradiol concentrations in women aged 40 years or older at diagnosis [9].

This prior study within the FMC represents the only published data on pregnancy hormone concentrations and breast cancer risk by hormone receptor status. Therefore, we investigated early pregnancy endogenous hormones and breast cancer risk in primiparous women by hormone receptor subtype (ER, PR) using the Northern Sweden Maternity Cohort (NSMC). In addition, we conducted an exploratory analysis of early pregnancy hormones and breast cancer risk by tumor androgen receptor (AR) status; to our knowledge, this is the first investigation of early pregnancy hormones and breast cancer risk by AR status.

## Methods

The NSMC was established in 1975 and is based at the Northern Sweden University Hospital, Umeå, Sweden. Details of the study and case–control selection have been published previously [11, 12]. Briefly, participants were enrolled from the four northern counties of Sweden while attending a maternity care unit run by the Swedish National Health Care System. These units provide prenatal and postnatal care free of charge to all pregnant women. Patients at these clinics provide a blood sample during the latter part of the first trimester or the early weeks of the second trimester to test for rubella antibodies. Remaining serum is stored at −20 °C and preserved in a central repository at the University Hospital in Umeå. The NSMC biological bank contains more than 150,000 serum samples from approximately 100,000 women.

To be eligible for this study, participants had to have provided a blood sample prior to 120 gestational days of pregnancy, during a singleton primiparous pregnancy resulting in a term birth (gestational age (GA) at delivery >37 to <43 weeks). Women aged 40 years or older at sample collection or with a prior cancer diagnosis (except nonmelanoma skin cancer) were excluded. Further, due to changes in sample handling which may affect hormone measurements, samples drawn after January 1, 1988 were excluded [11]. Invasive breast cancer cases (*n* = 223) were identified via linkage with the Swedish Cancer Registry; registration of newly diagnosed cancers is based on mandatory reporting; case ascertainment for breast cancer is estimated to be almost 100% [13]. Details of control selection have been published previously [12]. Briefly, cases and controls were matched 1:2; controls (*n* = 417) were selected among women alive and cancer-free (except nonmelanoma skin cancer) at the time of diagnosis of the corresponding case, with a blood sample available from a singleton primiparous pregnancy resulting in a term birth, and age < 40 years at sample collection. Controls were matched to cases on age at blood sampling (±6 months) and date of blood sampling (±3 months). Data on pregnancy and delivery characteristics were obtained from participants’ medical records.

### Laboratory assays

To ascertain hormone receptor status, all tumors were reevaluated by one pathologist (ET) to ensure tumor invasiveness in the analyzed tissue block; immunohistochemical assessments were performed by two pathologists (ET, HO). Paraffin blocks containing tumor tissue were gathered and new sections 4 μm thick were made. Immunohistochemical stains were performed on all cases using the following primary antibodies: estrogen receptor clone SP1, Ventana ready to use 790-4325; progesterone receptor clone IE2, Ventana ready to use 790-4296; and androgen receptor clone F39.4.1, Biogenex 1:50 MU256-UCE. All stains were performed on a Ventana XT platform using locally validated protocols. Immunohistochemical stains were evaluated as follows: brown nuclei were considered positive regardless of staining intensity. The percentage of positive tumor cells was registered. A cutoff value of 10% was used to define receptor positivity.

All serum hormone assays were conducted in the Department of Clinical Chemistry at Umeå University. Samples from cases and matched controls were analyzed in the same analytical batch alongside blinded quality control samples (6% of total samples). Sex steroids were quantified in two study phases (phase 1, *n* = 440; phase 2, *n* = 200) by high-performance liquid chromatography tandem mass spectrometry on an Applied Biosystems API4000 triple-stage quadrupole mass spectrometer. Sex hormone binding globulin (SHBG) was quantified with a solid-phase competitive chemiluminescence assay on an Immulite 2000 Siemens analyzer. Free estradiol and free testosterone were calculated using the formula of Södergård et al. [14]. Intrabatch coefficients of variation (CVs) ranged from 2% for progesterone to 16% for estrone; interbatch CVs ranged from 3% for estradiol to 13% for progesterone.

### Statistical analyses

Hormones were log_2_ transformed to improve the normality of the data, and to allow for an estimation of the effect of a doubling of hormone concentrations. To account for differences in hormone concentrations by study phase (concentrations presented in Additional file 1: Table S1) we centered concentrations from each of the two study phases at a mean of 0. Specifically, for each study phase, we calculated the mean concentration for each hormone and then subtracted this value from the observed concentration for each study participant within that study phase.

Hormone concentrations ≥ 3 standard deviations (SDs) from the mean were classified as outliers. No outliers were identified. The study population was categorized into tertiles, based on the study phase-specific distribution in study controls. Risk associated with a doubling of hormone concentration was assessed by modeling the log_2_-transformed hormones as continuous variables; the reported *p* for trend is from the model including the continuous log_2_-transformed hormone concentration.

We used conditional logistic regression to estimate odds ratios (ORs) and 95% confidence intervals (CIs). All models controlled for GA at blood collection; this was decided a priori given established associations between GA and hormone concentrations. Further, we observed changes in the OR of >10% comparing crude to GA-adjusted ORs for a subset of associations. The following factors were considered as potential covariates: number of previous pregnancies at blood collection (1 vs >1; includes women with prior spontaneous or induced abortion), maternal body mass index (BMI) at enrollment (kg/m^2^; continuous), current smoking (yes/no), placental weight (g), and the neonate’s birth weight (g). None of these factors changed the OR by more than 10%; therefore, these variables were not included in the final models. We further assessed the impact of adjusting for estradiol, in the models evaluating testosterone as the exposure, given that this has been shown to attenuate testosterone–breast cancer associations in previous studies (e.g., [6]).

We conducted analyses by hormone receptor status of the tumor (ER+/PR+ vs ER–/PR–, AR+, AR+/ER+/PR+), as well as analyses stratified by age at blood collection (<25 vs ≥25 years), age at diagnosis (<45 vs ≥45 years), lag-time between blood collection and diagnosis (<15 vs ≥15 years and <20 vs ≥ 20 years), number of pregnancies at blood collection (1 vs >1), and parity at diagnosis or index date (1 vs >1). Heterogeneity (*p*_het_) between ER+/PR+ and ER–/PR– breast cancer subtypes was assessed using a likelihood ratio test comparing models assuming the same association between the exposures and breast cancer overall to one assuming different associations by subtype. Interaction was tested by including a multiplicative interaction term in the models and evaluating the Wald *p* value.

All statistical tests are two-sided and considered statistically significant at *p* < 0.05. All analyses were conducted in SAS 9.3 (Cary, NC, USA).

## Results

Median age at blood donation in cases and controls was 26.5 (range: 17–39; matching factor; Table 1) and median GA at blood collection was approximately 10 weeks (cases: 72 days GA; controls: 70 days). The majority of women provided a blood sample during their first ever pregnancy (82% of cases; 78% of controls), and were multiparous at diagnosis or selection as a control (75% of cases; 81% of controls).

**Table 1:** Maternal and child characteristics in the Northern Sweden Maternity Cohort

Characteristic	Cases (*n* = 223)	Controls (*n* = 417)
Age at blood collection (years)	26.5 (17–39)	26.5 (17–39)
Gestational age at blood collection (days)	72 (36–116)	70 (37–120)
Children at diagnosis/selection as control		
1	55 (25%)	78 (19%)
2	120 (54%)	209 (50%)
3	48 (22%)	130 (31%)
Number of pregnancies at blood collection^a^		
1	182 (82%)	327 (78%)
2	35 (16%)	75 (18%)
3	6 (3%)	15 (4%)
Maternal weight^b^ (kg)	61 (39–89)	60 (37–127)
Maternal height^c^ (cm)	165 (147–183)	165 (144–180)
Current smoker	73 (33%)	121 (29%)
Child weight (g)	3470 (2300–4680)	3450 (2115–4770)
Case characteristics		
Age at diagnosis (years)	46.7 (25.5–63.8)	
Lag-time (years)	19.8 (2.7–30.5)	
Tumor characteristics		
ER status		
Positive	171 (77%)	
Negative	52 (23%)	
PR status		
Positive	157 (70%)	
Negative	66 (30%)	
AR status^d^		
Positive	77 (84%)	
Negative	15 (16%)	

Data presented as median (range) or *n* (%)

*AR* androgen receptor, *ER* estrogen receptor, *PR* progesterone receptor

^a^Number of pregnancies includes induced and spontaneous abortions

^b^Maternal weight missing for four cases

^c^Maternal height missing for 10 cases and 19 controls

^d^AR status available for 41% of cases

Higher early pregnancy testosterone and SHBG were associated with higher breast cancer risk (testosterone, third vs first tertile OR_(T3–T1)_: 1.46 (95% CI: 0.96–2.21), *p*_trend_ = 0.04; SHBG: 1.68 (1.05–2.70), *p*_trend_ = 0.13) (Table 2). Associations between testosterone (*p*_het_ = 0.05), free testosterone (*p*_het_ < 0.01), and progesterone (*p*_het_ = 0.01) and breast cancer differed by tumor hormone receptor status (Table 3). Testosterone and free testosterone were positively associated with ER+/PR+ disease and not associated with ER–/PR– disease (e.g., testosterone: ER+/PR+ OR_log2_: 1.57 (1.13–2.18); ER–/PR– OR_log2_: 0.82 (0.47–1.43)). Progesterone was inversely associated with ER+/PR+ disease (OR_log2_: 0.64 (0.41–1.00)) but not significantly associated with ER–/PR– disease (OR_log2_: 1.61 (0.77–3.36), *p*_het_ = 0.01). The association between progesterone and ER+/PR+ breast cancer was strengthened after adjustment for circulating estradiol concentrations (OR _log2_: 0.56 (0.35–0.91)).

**Table 2:** Early pregnancy endogenous hormones and breast cancer risk: Northern Sweden Maternity Cohort

		Tertile			*p*_trend_
		1	2	3	
Estradiol					
All women	Cases/controls	68/137	70/134	82/133	
	OR (95% CI)	Reference	1.06 (0.69–1.63)	1.29 (0.80–2.07)	0.15
ER+/PR+	Cases/controls	47/98	54/94	51/90	
	OR (95% CI)	Reference	1.16 (0.69–1.93)	1.11 (0.62–1.97)	0.60
Free estradiol					
All women	Cases/controls	67/127	78/126	65/121	
	OR (95% CI)	Reference	1.14 (0.74–1.75)	0.95 (0.59–1.54)	0.86
ER+/PR+	Cases/controls	48/90	54/83	41/85	
	OR (95% CI)	Reference	1.13 (0.68–1.90)	0.81 (0.45–1.44)	0.72
Estrone					
All women	Cases/controls	63/138	81/137	78/135	
	OR (95% CI)	Reference	1.27 (0.85–1.91)	1.25 (0.79–1.97)	0.45
ER+/PR+	Cases/controls	47/97	60/96	47/93	
	OR (95% CI)	Reference	1.25 (0.77–2.02)	0.96 (0.55–1.65)	0.79
Testosterone					
All women	Cases/controls	30/137	77/136	84/134	
	OR (95% CI)	Reference	1.29 (0.84–1.96)	1.46 (0.96–2.21)	0.04
ER+/PR+	Cases/controls	36/99	57/95	60/89	
	OR (95% CI)	Reference	1.69 (1.00–2.86)	1.94 (1.16–3.25)	0.01
Free testosterone					
All women	Cases/controls	73/128	57/127	81/122	
	OR (95% CI)	Reference	0.81 (0.52–1.25)	1.16 (0.73–1.83)	0.47
ER+/PR+	Cases/controls	44/97	41/79	59/84	
	OR (95% CI)	Reference	1.23 (0.72–2.12)	1.68 (0.96–2.94)	0.04
Progesterone					
All women	Cases/controls	74/138	80/134	67/134	
	OR (95% CI)	Reference	1.10 (0.72–1.68)	0.89 (0.56–1.40)	0.77
ER+/PR+	Cases/controls	55/84	55/99	43/99	
	OR (95% CI)	Reference	0.75 (0.44–1.27)	0.55 (0.31–0.99)	0.05
SHBG					
All women	Cases/controls	59/128	67/127	86/129	
	OR (95% CI)	Reference	1.21 (0.78–1.87)	1.68 (1.05–2.70)	0.13
ER+/PR+	Cases/controls	44/85	46/83	55/96	
	OR (95% CI)	Reference	1.08 (0.64–1.83)	1.18 (0.67–2.08)	0.70

Conditional logistic regression controlling for gestational age at blood collection (continuous)

*CI* confidence interval, *ER* estrogen receptor, *OR* odds ratio, *PR* progesterone receptor, *SHBG* sex hormone binding globulin

**Table 3:** Early pregnancy endogenous hormones and breast cancer risk for a doubling of hormone concentration, by ER/PR status: Northern Sweden Maternity Cohort

	Overall	ER–/PR–	ER+/PR+	*p*_het_
Estradiol	220/404	50/89	152/282	
	1.19 (0.94–1.50)	1.35 (0.78–2.32)	1.08 (0.81–1.43)	0.18
Free estradiol	210/374	49/86	143/258	
	1.02 (0.80–1.29)	1.09 (0.59–2.02)	0.95 (0.72–1.27)	0.31
Estrone	222/410	50/90	154/286	
	1.08 (0.89–1.30)	1.20 (0.77–1.88)	0.97 (0.78–1.22)	0.13
Testosterone	221/407	50/90	153/283	
	1.33 (1.02–1.74)	0.82 (0.47–1.43)	1.57 (1.13–2.18)	0.05
Free testosterone	211/377	49/87	144/260	
	1.08 (0.88–1.33)	0.66 (0.40–1.08)	1.31 (1.02–1.68)	<0.01
Progesterone	221/406	50/90	153/282	
	0.94 (0.67–1.34)	1.61 (0.77–3.36)	0.64 (0.41–1.00)	0.01
SHBG	212/384	49/89	145/264	
	1.19 (0.95–1.50)	1.47 (0.90–2.40)	1.05 (0.80–1.38)	0.10

Data presented as number of cases/controls and OR_log2_ (95% CI)

Conditional logistic regression controlling for gestational age at blood collection (continuous). *p*_het_ between ER+/PR+ and ER–/PR– breast cancer subtypes assessed using a likelihood ratio test comparing models assuming the same association between the hormones and breast cancer overall to one assuming different associations by subtype

*CI* confidence interval, *ER* estrogen receptor, *OR* odds ratio, *PR* progesterone receptor, *SHBG* sex hormone binding globulin

We cross-classified participants by circulating estradiol and progesterone concentrations to explore the joint effects of these hormones (i.e., estradiol/progesterone dichotomized at the median: low/low, low/high, high/low, high/high). In these models, the combination of high estradiol and low progesterone, relative to low concentrations of both, was associated with significantly higher risk of overall breast cancer (overall OR: 1.87 (1.11–3.16)) (Table 4). Results for ER+/PR+ disease were similar to those observed for overall breast cancer (ER+/PR+ OR: 1.81 (0.97–3.40); ER–/PR– OR: 1.42 (0.43–4.64)).

**Table 4:** Cross-classification of early pregnancy estradiol and progesterone and breast cancer risk: Northern Sweden Maternity Cohort

		Estradiol/progesterone			
		Low/low (<median/<median)	Low/high (<median/≥median)	High/low (≥median/<median)	High/high (≥median/≥median)
All women	Cases/controls	66/141	32/59	47/56	75/145
	OR (95% CI)	Reference	1.14 (0.67–1.94)	1.87 (1.11–3.16)	1.10 (0.95–1.76)
ER+/PR+	Cases/controls	47/99	22/48	33/37	50/101
	OR (95% CI)	Reference	0.92 (0.49–1.75)	1.81 (0.97–3.40)	0.98 (0.56–1.71)
ER–/PR–	Cases/controls	15/33	6/7	12/16	17/33
	OR (95% CI)	Reference	1.63 (0.46–5.79)	1.42 (0.43–4.64)	0.80 (0.29–2.15)

Conditional logistic regression controlling for gestational age at blood collection (continuous). Cases with reported ER+/PR– and ER–/PR+ tumors included in analysis of “all women”

*CI* confidence interval, *ER* estrogen receptor, *OR* odds ratio, *PR* progesterone receptor

We conducted exploratory analyses by tumor AR status (AR+ and AR+/ER+/PR+); data were available for 41% of cases. Overall, the baseline and case characteristics of cases with and without AR status available were similar (Additional file 1: Table S2). None of the investigated hormones were associated with breast cancer risk when analyses were restricted to AR+ or AR+/ER+/PR+ tumors (Additional file 1: Table S3). The association between testosterone and ER+/PR+ disease was positive, but was attenuated and not statistically significant, in the subset of ER+/PR+ cases with AR data (all ER+/PR+ cases: *n* = 153, OR_log2_: 1.57 (1.13–2.18); ER+/PR+ cases with AR data: *n* = 63, OR_log2_: 1.32 (0.78–2.25)).

In analyses stratified by age at diagnosis, testosterone was positively associated with risk in women with age at diagnosis ≥ 45 years (*n* = 135 cases, OR_log2_: 1.48 (1.06–2.05)), but not associated with risk in women diagnosed at age < 45 years (*n* = 86 cases, OR _log2_: 1.08 (0.68–1.71)); the difference was not statistically significant (*p*_het_ = 0.28). The association between testosterone and breast cancer risk was strengthened after adjustment for estradiol concentrations (ER–/PR- OR_log2_: 0.64 (95% CI: 0.33–1.25); ER+/PR+ OR_log2_: 1.64 (1.15–2.33)).

Results stratified by age at blood collection (<25 vs ≥25 years), age at diagnosis (<45 vs ≥45 years), time from blood collection to diagnosis (<20 vs ≥20 years), gravidity at blood collection (1 vs >1), and parity at diagnosis or selection as a control (1 vs >1) were similar (data not shown). We observed significant heterogeneity for free testosterone when comparing associations among participants with <15 and ≥15 years between blood collection and diagnosis (*p* = 0.04; *n* = 42 cases diagnosed < 15 years after blood collection); however, the individual effect estimates were not statistically significant (<15 years: 0.62 (0.35–1.10); ≥15 years: 1.20 (0.95–1.50)).

## Discussion

We expanded the limited prior literature on endogenous hormones in pregnancy and breast cancer risk by hormone receptor status. We observed an inverse association between early pregnancy progesterone and subsequent risk of ER+/PR+ breast cancer. Further, we observed positive associations between early pregnancy testosterone and free testosterone and breast cancer, predominantly in ER+/PR+ tumors. Early pregnancy estrogens alone were not associated with breast cancer, but high estradiol in the context of low progesterone was associated with higher risk, relative to low concentrations of both hormones. We observed no significant associations between endogenous hormones and ER–/PR– or AR+ disease.

Estrogens and progesterone increase several-fold during pregnancy relative to prepregnant concentrations [15, 16]; these hormones are of placental origin. Estradiol and progesterone have well established roles in breast development [17, 18], and data from animal models suggest that mimicking the hormonal milieu of pregnancy with estradiol and progesterone confers similar protection against breast cancer as is conferred by pregnancy [2]. Progesterone is essential for normal lobular–alveolar development and differentiation in the breast [19]; our study quantified progesterone in early pregnancy, when the breast is undergoing proliferation and the early stages of pregnancy-associated differentiation.

Sex steroid hormones in pregnancy and breast cancer risk in the mother have been investigated previously in three studies nested within two populations [8–10]. Peck et al. investigated third-trimester hormones in the Child Health and Development Study (CHDS), observing a suggestive inverse association between progesterone and breast cancer risk (*n* = 194 cases; OR, extreme deciles: 0.49 (0.2–1.1); *p*_trend_ = 0.08). A positive association was observed between estrone and disease risk (OR, extreme deciles: 2.5 (1.0–6.1); *p*_trend_ = 0.12). Pregnancy estradiol and estriol were not associated with risk [8]. No association was observed between early pregnancy sex steroids and overall breast cancer in the most recent study in the FMC (*n* = 1199 cases) [9]. However, estradiol was positively associated with breast cancer diagnosed before age 40 (fourth vs first quartile OR: 1.60 (1.07-2.39)) and suggestively inversely associated with breast cancer diagnosis at age 40 years or older (fourth vs first quartile OR: 0.71 (0.51-1.00); *p*_het_ < 0.01). Associations among women younger than age 40 years at diagnosis were only observed for ER–/PR– tumors. In the FMC, progesterone was associated with increased risk of ER–/PR– disease among women diagnosed before age 40 years, but not associated with risk among women aged 40 years or older.

In line with findings from the CHDS, we observed an inverse association between progesterone and breast cancer risk in the current study, although this was restricted to ER+/PR+ tumors. Blood collection in the current study was at median 10 weeks GA, in contrast to mean of 34.5 weeks in the CHDS. Progesterone concentrations are modestly correlated across trimesters of a single pregnancy (Spearman correlations: first and second trimesters, *r* = 0.63; first and third trimesters, *r* = 0.39; second and third trimesters, *r* = 0.64) [16], suggesting that one measure in early pregnancy may not represent late pregnancy concentrations, particularly considering first and third trimester concentrations. Therefore, both early pregnancy progesterone, as measured here, and late pregnancy progesterone, as measured in the CHDS, may impact subsequent breast cancer risk.

We observed no association between estradiol and breast cancer overall risk in the current study, with the exception of an increased risk of disease in women with relatively high estradiol (above median) and low progesterone (below median), as compared to women with low concentrations of both hormones; this increase in risk was evident for both hormone receptor-positive and receptor-negative disease. Experimental data from animal models suggest that both estradiol and progesterone may be necessary to induce the long-term protective effect of pregnancy, although results differed based on the experimental model [2, 20]. The association between breast cancer and cross-classified estradiol and progesterone was not described in the previous investigations in pregnant women, nor, to our knowledge, in epidemiologic studies in premenopausal women. However, high concentrations of circulating endogenous estrogens after menopause—a period characterized by physiologically low circulating progesterone concentrations— are consistently associated with increased risk of breast cancer [5–7].

Given the divergent association between estradiol and breast cancer risk in analyses stratified by age at diagnosis in the FMC, we evaluated risk stratified by age at diagnosis in this study (<45 vs ≥45 years). Results were similar in both age groups for estrogens and progesterone, while testosterone was more strongly associated with breast cancer diagnosed at age 45 years or older. Given the age distribution in our cohort, we were unable to evaluate risk using the same age thresholds as the FMC (i.e., only 30 cases in our population were diagnosed prior to age 40 years). The FMC population was somewhat older at first birth than the NSMC study population and due to technical considerations (i.e., restriction of the study population to women providing blood samples prior to 1988 and longer follow-up), women in the NSMC were diagnosed at an older age (median age at diagnosis: FMC = 41.2 years; NSMC = 46.7 years) and after longer lag-time between pregnancy and cancer diagnosis (median lag-time: FMC = 10.9 years; NSMC = 19.8 years). Therefore, our findings from the NSMC may pertain to the long-term impact of early pregnancy hormones and breast cancer risk, whereas the FMC results may better describe risk associated with more proximate exposure to pregnancy hormones.

In contrast to estrogens and progesterone, androgens increase gradually across pregnancy, approximately doubling from preconception to the third trimester [15, 16]. Androgens are produced by the ovary and maternal adrenal cortex as well as the adrenal glands and liver of the fetus [21]. Androgens are relatively stable from prepregnancy to early pregnancy, and the androgens quantified in our study are likely representative of circulating premenopausal androgen concentrations. Epidemiologic data consistently show a positive association between androgens and breast cancer risk, in both premenopausal [3, 4] and postmenopausal [5–7] women. This may be due to a direct androgen effect, or may be a result of conversion of androgens to estrogens in breast tissue via aromatase; aromatase is expressed in both normal and malignant tissue [22].

We observed higher risk of ER+/PR+ breast cancer risk with higher circulating testosterone concentrations in the current study. In the only prior study on androgens in pregnancy and breast cancer risk, in the FMC [9], testosterone was positively associated with risk of ER–/PR– tumors in the subgroups of women diagnosed younger than age 40 or with first birth younger than age 30; testosterone was not associated with breast cancer overall or in the ER+/PR+ subgroup. As with estrogens and progesterone, the divergent findings between the current study and results from the FMC may be due to the different age distributions and interval between pregnancy and breast cancer diagnosis in the two study populations.

To our knowledge, our study is the first investigation of early pregnancy hormones and maternal breast cancer risk by androgen receptor status. Experimental data suggest that crosstalk between the ER and AR results in impeded receptor signaling of both receptors, thus inhibiting hormone-related growth and proliferation [23]. Further, epidemiologic data show ER+/AR+ tumors have better prognosis that ER+/AR– tumors [23]. We observed a positive association between testosterone and ER+/PR+ disease in all cases. The associations between testosterone and ER+/PR+/AR+ and ER+/PR+ breast cancer risk, among the subset of women with AR data, were similar (among women with AR data: ER+/PR+ OR_log2_: 1.32; ER+/PR+/AR+ OR_log2_: 1.36), suggesting the AR may not play an important role in this context.

Our study has important strengths and limitations. Blood samples were collected and stored using standardized procedures. However, samples are stored at a relatively warm temperature (−20 °C). Estradiol and SHBG concentrations were weakly correlated with storage time (estradiol: *r* = 0.19, *p* < 0.01; SHBG: *r* = −0.20, *p* < 0.01), as were free estradiol (*r* = 0.19, *p* < 0.01) and free testosterone (*r* = 0.13, *p* = 0.01). Cases and controls were carefully matched for date of blood collection, therefore the weak correlations observed between hormone concentrations and storage time should not impact our results. Hormones change systematically in early pregnancy with GA. We accounted for these changes by adjusting for GA in regression models. An alternative approach would be to use regression residuals. In our study, results adjusting for GA were similar to those using regression residuals. Finally, sample size was limited for analyses of ER–/PR– tumors, and AR data were only available for a subset of cases.

## Conclusions

We observed an inverse association between early pregnancy progesterone and subsequent risk of ER+/PR+ breast cancer as well as a positive association between early pregnancy testosterone and free testosterone and breast cancer, predominantly in ER+/PR+ tumors. This investigation adds to the limited literature on early pregnancy hormones and breast cancer risk in the mother. As with prior studies, our study investigated whether relatively high vs lower concentrations of hormones were associated with subsequent risk of breast cancer among parous women. Further studies investigating novel markers are necessary to better characterize the relationship between early pregnancy hormones and maternal breast cancer among this population.

## Additional file

Additional file 1:
**Table S1.** Presenting early pregnancy endogenous hormone concentrations (median (range) by study phase in the Northern Sweden Maternity Cohort, **Table S2.** presenting maternal and child characteristics by AR status availability among cases (median (range) or *n* (%)) in the Northern Sweden Maternity Cohort, and **Table S3.** presenting early pregnancy endogenous hormones and breast cancer risk for a doubling of hormone concentration, by AR+ and AR+/ER+/PR+ cases in the Northern Sweden Maternity Cohort. (DOCX 25 kb)

## Abbreviations

AR: Androgen receptor
BMI: Body mass index
CHDS: Child Health and Development Study
CI: Confidence interval
CV: Coefficient of variation
ER: Estrogen receptor
FMC: Finnish Maternity Cohort
GA: Gestational age
NSMC: Northern Sweden Maternity Cohort
OR: Odds ratio
PR: Progesterone receptor
SD: Standard deviation
SHBG: Sex hormone binding globulin
