# Supplementary material for: Early pregnancy sex steroids during primiparous pregnancies and maternal breast cancer: a nested case–control study in the Northern Sweden Maternity Cohort
Source: Breast Cancer Res. 2017 Jul 18;19:82. doi: 10.1186/s13058-017-0876-8 (PMC5516370; doi:10.1186/s13058-017-0876-8)
Supplement: Additional file 1: — Table S1. Presenting early pregnancy endogenous hormone concentrations (median (range) by study phase in the Northern Sweden Maternity Cohort, Table S2. presenting maternal and child characteristics by AR status availability among cases (median (range) or n (%)) in the Northern Sweden Maternity Cohort, and Table S3. presenting early pregnancy endogenous hormones and breast cancer risk for a doubling of hormone concentration, by AR+ and AR+/ER+/PR+ cases in the Northern Sweden Maternity Cohort. (DOCX 25 kb) [file 13058_2017_876_MOESM1_ESM.docx]

| **Table S1. Early pregnancy endogenous hormone concentrations (median (range) by study phase: Northern Sweden Maternity Cohort (n=640)** | | |  |
| --- | --- | --- | --- |
| **Hormone Concentrations** | **Cases** | **Controls** | |
| **PHASE 1** | 153 | 287 | |
| Estradiol (ng/mL) | 2.12 (0.45-9.0) | 1.91 (0.35-9.8) | |
| Free Estradiol (pmol/L) | 116 (27-573) | 116 (23-514) | |
| Estrone (ng/mL) | 0.76 (0.09-4.8) | 0.74 (0.12-4.9) | |
| Testosterone(ng/mL) | 0.84 (0.30-3.2) | 0.83 (0.25-4.1) | |
| Free Testosterone (pmol/L) | 21 (6.1-210) | 21 (4.2-102) | |
| Progesterone (ng/mL) | 24 (8.8-63) | 25 (5.3-60) | |
| SHBG (nmol/L) | 129 (23-347) | 117 (20-321) | |
|  |  |  | |
| **PHASE 2** | 70 | 130 | |
| Estradiol (ng/mL) | 1.75 (0.27-11.4) | 1.53 (0.17-11.5) | |
| Free Estradiol (pmol/L) | 101 (8.6-436) | 89 (11-430) | |
| Estrone (ng/mL) | 0.61 (0.11-7.2) | 0.55 (0.09-4.8) | |
| Testosterone(ng/mL) | 0.71 (0.24-3.2) | 0.66 (0.18-2.6) | |
| Free Testosterone (pmol/L) | 21 (2.3-91) | 16 (1.8-84) | |
| Progesterone (ng/mL) | 20 (7.3-67) | 20 (8.3-64) | |
| SHBG (nmol/L) | 129 (23-441) | 114 (21-1298) | |

| **Table S2. Maternal and child characteristics by AR status availability among cases (median (range) or n(%)) in the Northern Sweden Maternity Cohort (n=223)** | | |
| --- | --- | --- |
| **Characteristic** | **AR Status  Available  (n=92)** | **AR Status  Missing  (n=131)** |
| Age at blood collection, years | 26.4 (17-37.7) | 26.5 (18.3-39) |
| Gestational age at blood collection, days | 70.5 (36-116) | 73.0 (38-114) |
| Children at diagnosis/selection as control |  |  |
| 1 | 19 (21%) | 36 (27%) |
| 2 | 52 (57%) | 68 (52%) |
| 3 | 21 (23%) | 27 (21%) |
| Number of pregnancies at blood collection* |  |  |
| 1 | 69 (75%) | 113 (86%) |
| 2 | 20 (22%) | 15 (11%) |
| 3 | 3 (3%) | 3 (2%) |
| Maternal Weight**, kg | 62 (39-89) | 60 (45-87) |
| Maternal Height**, cm | 166 (147-177) | 164 (150-183) |
| Current Smoker | 31 (34%) | 42 (32%) |
| Child Weight, grams | 3495 (2300-4260) | 3400 (2115-4770) |
|  |  |  |
| **Case Characteristics** |  |  |
| Age at diagnosis, years | 45.9 (25.5-61.7) | 46.9 (27.7-63.8) |
| Lag time, years | 19.6 (3.0-29.7) | 19.9 (2.7-30.5) |
| Tumor characteristics |  |  |
| ER status |  |  |
| Positive | 70 (76%) | 101 (77%) |
| Negative | 20 (24%) | 30 (23%) |
| PR status |  |  |
| Positive | 64 (70%) | 93 (71%) |
| Negative | 28 (30%) | 38 (29%) |
| AR Status |  |  |
| Positive | 77 (84%) | -- |
| Negative | 15 (16%) | -- |
| *Number of pregnancies includes induced and spontaneous abortions;  ** AR available subgroup: maternal weight missing for 1 case; maternal height missing for 3 cases; AR missing subgroup: maternal weight missing for 3 cases; maternal height missing for 7 cases | | |

| **Table S3. Early pregnancy endogenous hormones and breast cancer risk for a doubling of hormone concentration, by AR+ and AR+/ER+/PR+ cases: Northern Sweden Maternity Cohort*** | | | | |
| --- | --- | --- | --- | --- |
|  |  | **AR+** |  | **AR+/ER+/PR+** |
|  |  | cases/controls OR_log2_ (95% CI) |  | cases/controls OR _log2_ (95% CI) |
| **Estradiol** |  | 76/139 |  | 60/112 |
|  |  | 1.06 (0.72 - 1.55) |  | 1.18 (0.77 - 1.80) |
|  |  |  |  |  |
| **Free Estradiol** |  | 72/129 |  | 57/103 |
|  |  | 0.95 (0.65 - 1.40) |  | 1.00 (0.65 - 1.54) |
|  |  |  |  |  |
| **Estrone** |  | 77/142 |  | 61/114 |
|  |  | 1.08 (0.78 - 1.49) |  | 1.08 (0.75 - 1.57) |
|  |  |  |  |  |
| **Testosterone** |  | 77/141 |  | 61/113 |
|  |  | 1.15 (0.72 - 1.84) |  | 1.36 (0.80 - 2.32) |
|  |  |  |  |  |
| **Free Testosterone** |  | 72/129 |  | 57/103 |
|  |  | 0.99 (0.66 - 1.51) |  | 1.24 (0.77 - 1.99) |
|  |  |  |  |  |
| **Progesterone** |  | 77/142 |  | 61/114 |
|  |  | 0.81 (0.45 - 1.48) |  | 0.59 (0.28 - 1.20) |
|  |  |  |  |  |
| **SHBG** |  | 72/132 |  | 57/105 |
|  |  | 1.16 (0.79 - 1.72) |  | 1.01 (0.66 - 1.56) |
| Conditional logistic regression controlling for gestational age at blood collection (continuous).  *AR status available for 41% of cases | | | | |
